# A Survey Study on Gastrointestinal Parasites of Stray Cats in Northern Region of Nile Delta, Egypt

**DOI:** 10.1371/journal.pone.0020283

**Published:** 2011-07-08

**Authors:** Reda E. Khalafalla

**Affiliations:** Department of Parasitology, Faculty of Veterinary Medicine, Kafrelsheikh University, Kafrelsheikh, Egypt; Weill Cornell Medical College, United States of America

## Abstract

A survey study on gastrointestinal parasites in 113 faecal samples from stray cats collected randomly from Kafrelsheikh province, northern region of Nile delta of Egypt; was conducted in the period between January and May 2010. The overall prevalence was 91%. The results of this study reported seven helminth species: *Toxocara cati* (9%), *Ancylostoma tubaeforme* (4%), *Toxascaris leonina* (5%), *Dipylidium caninum* (5%), *Capillaria* spp. (3%), *Taenia taeniformis* (22%) and *Heterophyes heterophyes* (3%), four protozoal species: *Toxoplasma gondii* (9%), *Sarcocyst* spp. (1%), *Isospora* spp. (2%) and *Giardia* spp. (2%) and two arthropod species; *Linguatula serrata* (2%) and mites eggs (13%).

The overall prevalence of intestinal parasites may continue to rise due to lack of functional veterinary clinics for cat care in Egypt. Therefore, there is a need to plan adequate control programs to diagnose, treat and control gastrointestinal parasites of companion as well as stray cats in the region.

## Introduction

Gastrointestinal parasites are the main causes of morbidity in domestic cats [1]. In Egypt and other parts of the world these parasites cause great public health problems.

Several factors affect the frequency of a species of parasite in a population. The prevalence of intestinal parasites can vary due to geographical region; presence of veterinary care; habits of the local animal populations; season of the year and the cat population composition. Several epidemiological surveillance studies reported that feral/stray cats present high frequency of parasites [2], [3], [4], [5].

In Egypt, little is known about the parasites of cats. This knowledge allows for improved explanations as to the distribution of parasitism and its significance to the health of humans and animals inhabiting the area under study. So that, the aim of this study is to determine the parasites of stray cats inhabiting the Nile Delta region of Lower Egypt.

Nile Delta of Egypt is that territory situated from south to north between Cairo and the Mediterranean Sea, respectively and from west to east between the Rosetta branch and the Damietta branch of the Nile River, respectively. The population density is very high and estimated by 34 million inhabitants over 25,000 km^2^, i.e., with 1,360 residents per km^2^ (Cairo population is not counted) [6].

The Nile Delta territory is characterized by a moderate climate. During summer it is moderately hot and dry where the temperature is ranged between 25–35°C while during winter its climate is warm and scanty to moderate rainy. Temperatures range between highs of 35 to 40°C during June to August, and lows of 5 to 10°C between December and January [6].

## Materials and Methods

Over the period between January and May 2010, 113 fecal samples of stray cats were collected in a weekly pattern from different sandy spots representing Kafrelsheikh province, defined as the northern part of the Nile Delta region of Egypt. Stray cats could not be caught and therefore could not be identified as to age, sex and breed. They were observed as closely as possible depositing and burying their feces in separate holes in sandy spots. Collection of the fecal samples determined that some samples were freshly deposited whereas others were not. Approximately 100 gm of cat feces were collected from individual holes and the remainder discarded hygienically.

All fecal samples were initially examined macroscopically for the presence of tapeworm proglottids or nematodes. Flotation centrifugation methods were applied using zinc sulphate and saturated salt solution (specific gravity 1.2) as described [7], [8], [9]. Identification of parasite species was performed based on egg and cyst morphology for the well documented species [10].

## Results and Discussion

**Figure 1:**
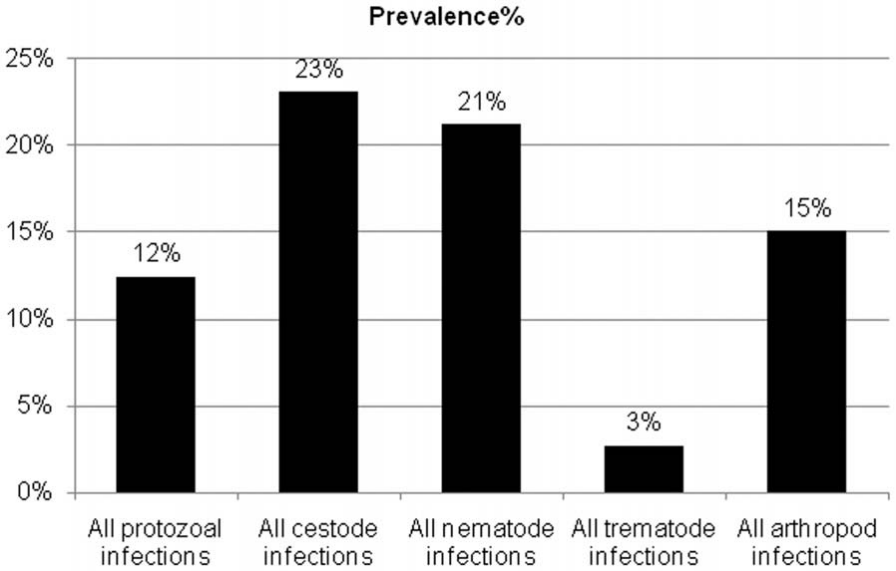
The overall prevalence of different infections in stray cats in northern region of Nile Delta, Egypt.

**Figure 2:**
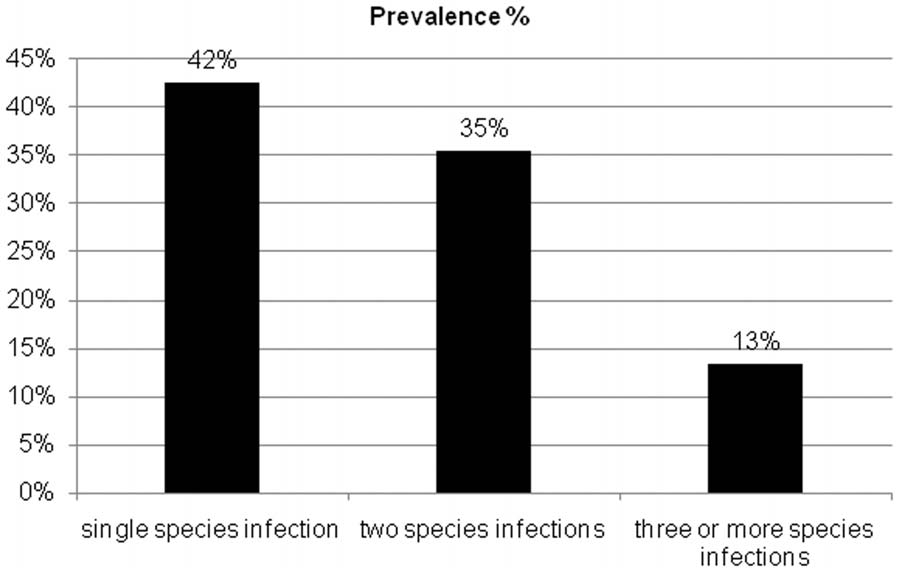
The prevalence of single species, two species and poly-infections (three or more species) in stray cats in northern region of Nile Delta, Egypt.

Description of the fecal parasite infections indicated that the overall infection rate was 91%. The individual prevalence of infections is shown in Table 1. The positively infected samples were infected with protozoa (12%), cestodes (23%), nematodes (21%), trematodes (3%) and arthropods (15%, Table 1 and Figure 1). Figure 2 and Table 2 present the type of the infection as 42%, 35% and 13% were infected with single (mono-infection), two to three (poly-infection) or more species of endoparasites, respectively.

**Table 1:** Prevalence of gastrointestinal parasites of stray cats in Kafrelsheikh province of Nile Delta region in Egypt (*n = 113* fecal samples).

parasites	Infected samples *(n = 113)*	Prevalence%
*Toxoplasma gondii*	*10*	*9%*
*Isospora* spp.	2	*2%*
*Giardia* spp.	2	*2%*
*Sarcocyst* spp.	1	*1%*
**All protozoal infections**	**14**	12%*
*Taenia taeniformis*	25	*22%*
*Dipylidium caninum*	6	5%
**All cestode infections**	**26**	23%*
*Toxocara cati*	10	*9%*
*Toxascaris leonina*	6	*5%*
*Ancylostoma tubaeforme*	5	*4%*
*Capillaria* spp.	3	*3%*
**All nematode infections**	**24**	21%*
*Heterophyes heterophyes*	3	*3%*
**All trematode infections**	**3**	3%*
Mites eggs	15	*13%*
*Liguatula serrata*	2	*2%*
**All arthropod infections**	**17**	15%*

*The total for each type (e.g.; Cestodes, Protozoa, etc) is sometimes lesser than the sum of individual infections.

**Table 2:** Types of different mixed infections with gastrointestinal parasites of stray cats in Kafrelsheikh province of Nile Delta region in Egypt (*n = 113* fecal samples).

mixed infection	prevalence (*n = 113*)	%
single species infection	48	42%
two species infections	40	35%
three or more species infections	15	13%
All infections	103	91%

The reported parasites were seven helminth species: *T. cati* (9%), *A. tubaeforme* (4%), *T. leonina* (5%), *D. caninum* (5%), *Capillaria* spp. (3%), *T. taeniformis* (22%) and *H. heterophyes* (3%), four protozoal species: *T. gondii* (9%), *Sarcocyst* spp. (1%), *Isospora* spp. 2% and *Giardia* spp. (2%) and two arthropod species; *Linguatula serrata* (2%) and mites eggs (13%; Table 1).

This study estimates a 91% prevalence of intestinal parasites in stray cats, and this figure is in general agreement with published reports of stray cats in northern Iran (90% prevalence; [11]); mid-Ebro Valley, Spain (90%; [5]); and Rio de Janeiro (90%; [12], [13]. However, comparison of the present study with published surveys indicated that great differences in prevalence were observed for particular parasite species; perhaps due to regional, environmental or climatic variations.

*T. taeniformis* was the dominant tapeworm reported in examined fecal samples of stray cats in Nile Delta of Egypt with a prevalence rate of 22% which is lower than that reported in Doha, Qatar (74%, [14]). However, it is more or less in the same range as that recorded in Cairo, Egypt (30%, [15]) and that in Iran (18%, [16]). While the prevalence rate of *T. taeniformis* in the current research is higher than that recorded in Jordan (3.8%, [16]) and in Iran (12% [17]).

*D. caninum* was encountered with low prevalence (5%) in comparison with other surveys. For example, *D. caninum* was harboured in 51% and 45% of the wildcats, *Felis catus,* necropsied in studies performed in Britain [18] and Egypt [15], respectively.

*T. cati* was found to be the frequent nematode eggs in the current study, however, the overall *T. cati* prevalence was relatively low (9%) in comparison with the prevalences encountered in Denmark (79%, [19]), in Spain (55%, [2]), in Greece (67%, [19]) and in England (53%, [5]).

*Ancylostoma tubaeforme*, *T. leonina* and *Capillaria* spp. were the other nematode species found in the present survey, with lower prevalences. For example, *A. tubaeforme,* in other studies, the estimated prevalences were 40% in Israel [20], 39.5% in Belgium [21] and 41% in the Republic of South Africa [22].

Mite eggs and sometimes mites larvae were found in 13% of examined fecal samples as well *Linguatula serrata* larvae were identified only in two samples (2%). In the present study, mite infection in the stray cats was evident and due to the cat's grooming habits, the mite eggs were swallowed and dropped with feaces.

In the current study, the all protozoan infections recovered was 12% which included *T. gondii* (9%), *Isospora* spp. (2%), *Giardia* spp. (2%) and *Sarcocystis* spp. (1%). The most dominant protozoal infection was *T. gondii* in stray cats recorded in the present study was generally within the reported results from the Middle East which revealed a range of Toxoplasmosis in stray and domestic cats from 12.5% to 78.1% [16], [23], [24], [25].

### Conclusion

High prevalence rate of cats with a wide range of parasitic organisms in the studied area suggests that inhabitants face risk of parasitic infections through contact with infected cats and their excretion. Therefore, both animal and human health education are recommended in the developed communities. As well the veterinarians and physicians should play an important role in increasing the degree of awareness of feline zoonotic parasites, which could be helpful to prevent or minimise zoonotic transmission.
